# PhamClust: a phage genome clustering tool using proteomic equivalence

**DOI:** 10.1128/msystems.00443-23

**Published:** 2023-10-04

**Authors:** Christian H. Gauthier, Graham F. Hatfull

**Affiliations:** 1 Department of Biological Sciences, University of Pittsburgh, Pittsburgh, Pennsylvania, USA; Los Alamos National Laboratory, Los Alamos, New Mexico, USA

**Keywords:** bacteriophages, genome comparison

## Abstract

Bacteriophage comparative genomics is complex due to the mosaic nature of the genomes, and an underlying continuum of diversity confounds the identification of clear taxonomic boundaries. Nucleotide sequence comparison methods have been described for phage taxonomy, but they are computationally intensive and scale poorly as the number of sequenced phage genomes increases. Here, we describe PhamClust as a new bioinformatic approach for grouping phages according to their inter-genome relatedness. PhamClust calculates a proteomic equivalence quotient (PEQ) for each pair of phages based on amino acid sequence identity for those genes that are shared among phages. PEQ values span from 0% (no shared genes) to 100% (all genes shared at 100% identity), and using a large mycobacteriophage genome data set, we show that two-step clustering down to a PEQ of 25% constructs genome groupings (clusters) closely mirroring those constructed manually over time, with the differences attributable to historically arising incongruities rather than illogicalities in PhamClust. PEQ values can also faithfully divide clusters into subclusters, although the relationships are highly heterogeneous, with different PEQ values needed for the subdivision of different clusters. PhamClust can be used to assort any group of phages, including the RefSeq phage collection.

**IMPORTANCE:**

Bacteriophage genomes are pervasively mosaic, presenting challenges to describing phage relatedness. Here, we describe PhamClust, a bioinformatic approach for phage genome comparisons that uses a new metric of proteomic equivalence quotient for comparative genomics. PhamClust reliably assorts genomes into groups or clusters of related phages and can subdivide clusters into subclusters. PhamClust is computationally efficient and can readily process thousands of phage genomes. It is also a useful analytic tool for exploring the different types of inter-genome relatedness characteristic of phages in different clusters.

## INTRODUCTION

Bacteriophages are the most abundant and genetically diverse biological entities in the biosphere (1). Like bacteria, their evolution is shaped by both vertical and horizontal modes of inheritance, but pervasive horizontal genetic exchange (HGE) has given rise to highly mosaic phage genomes (2, 3). However, the contribution of HGE differs depending on phage host, lifestyle, and genome (4). In general, temperate phages engage in greater levels of HGE than lytic phages, perhaps reflecting recombination events occurring when they are integrated as chromosomal prophages (4). Nonetheless, pervasive genomic mosaicism presents a substantial challenge to taxonomic strategies for grouping phages according to their genomic relationships, and whole genome phylogenies are often amalgams of the many different and distinct phylogenies of the composite genes (5). Furthermore, comparison of a large number of phages isolated on *Mycobacterium smegmatis* suggests that there is an underlying continuum of phage genetic diversity, and assortment into groups reflects differences in prevalence and sampling, rather than rigid biological barriers to genetic exchange (6).

Comparative analysis of mycobacteriophage genomes illustrates many of the challenges in representing phage genetic relationships. Over 2,200 genomes of individual mycobacteriophages have been sequenced and display substantial overall diversity (7). When a relatively small number of genomes had been sequenced, it was simple to group them into “clusters” based on overall DNA sequence similarity (8). As more genome sequences became available, there was evident heterogeneity within clusters, and this variation could be represented by division into subclusters, largely based on average nucleotide identity (ANI) comparisons. However, as the number of genomes increased, the threshold parameters reflecting these subcluster divisions appeared to be non-universal, and to vary for different genomic clusters (8, 9). The same phenomenon has been described for *Enterobacteriaceae* phages (10).

Nucleotide sequence comparisons are computationally intensive and have limitations when comparing highly mosaic genomes. VIRIDIC has been proposed as a nucleotide-based system for phage taxonomy but it is computationally expensive, limiting the number of genomes that can be readily analyzed (11). Several alternative methods for approximating phage taxonomy have been described, including shared gene content analysis (4) facilitated by assortment of phage genes into groups (phams or phamilies) using BLASTP (12), kClust (6, 12) or more recently MMseqs2 (13, 14), and determination of shared phams (4). Similar gene content-based methods have been described for comparison of bacterial genomes including calculation of the percentage of conserved proteins (POCP) (15), alignment fraction (AF) of genes (16), and proteomic approaches (17, 18). These types of comparisons are generally computationally inexpensive for large sets of genomes because they do not require orthologous gene alignment; therefore, runtime is essentially constant with respect to genome size. These alignment-free metrics are poorly effective at distinguishing genomes that are less impacted by HGE, but alignment-based approaches such as ANI or average amino acid identity (AAI) (19) perform similarly poorly when comparing very distantly related genomes that primarily share horizontally transferred genes.

Currently, the ~2,200 sequenced mycobacteriophage genomes have been sorted into 31 clusters (Clusters A, B, C, etc.), 12 of which are divided into subclusters (Subclusters A1, A2, A3, etc.), and there are seven “Singletons,” each of which has no close relatives (20, 21). Cluster sizes vary enormously, with over 740 Cluster A phages, but fewer than five each for Clusters U, V, X, Y, Z, AA, AB, AC, AD, and AE (https://phagesdb.org). Recently, it has been shown that some *Mycobacterium* genomes are replete with integrated prophages, with over 1,700 new unique prophage sequences that are diverse and generally not closely related to the *M. smegmatis* phages (22). To facilitate integration of the phage and prophage data sets and simplify computationally efficient exploratory analyses of inter-genomic relatedness, we have developed PhamClust, a tool that calculates an index we refer to as the proteomic equivalence quotient (PEQ), which provides a global estimate of genome similarities. We have determined the PEQ values which most closely reconstruct the extant cluster and subcluster groupings and show that PhamClust can efficiently process over 4,000 phage and prophage genomes. PhamClust also offers an analytical tool for illustrating different genomic relationships within different clusters and subclusters, an important consideration for phage taxonomy. PhamClust can be applied to any set of phage genomes, including the GenBank RefSeq phage data set.

## MATERIALS AND METHODS

### Programming and availability

PhamClust is a command-line Python program compatible with Python 3.6 and above. It uses the Python bindings for the PARASAIL alignment library (23) to efficiently compute global alignments between orthologous genes shared by any pair of genomes. Sklearn (https://pypi.org/project/scikit-learn) is used to perform hierarchical clustering based on calculated pairwise genomic distances, and Plotly (https://pypi.org/project/plotly) is used to generate heatmaps useful for visualizing the structure of resulting clusters and subclusters. PhamClust can be obtained from GitHub (https://github.com/chg60/phamclust) or installed from PyPI (https://pypi.org/project/phamclust).

### Description of command-line arguments

The command-line interface to PhamClust exposes several variables that can be used to alter the default behavior of PhamClust. These arguments, their descriptions, and default values can be seen by invoking PhamClust with the “--help” argument or visiting the documentation on GitHub but are also briefly described here. Users can choose between several metrics (see Pairwise genomic similarity calculations, below) to calculate intergenomic similarities as well as changing the thresholds and hierarchical clustering linkage types used for clustering and subclustering. Subclustering can be turned off entirely or set to be used only on clusters that include at least some target number of genomes. Finally, users can decide how many central processing unit (CPU) cores to leverage and whether to retain temporary files after the run completes (recommended for repeated runs, for example, to optimize alternative sets of suitable clustering thresholds).

### Data

The pdm_utils toolkit (24) was used to retrieve the “Actino_Draft” database (version 465) (containing sequenced and annotated Actinobacteriophages) from http://databases.hatfull.org, and to export all 2,121 mycobacteriophage proteomes as FASTA files of amino acid sequences. PhaMMseqs (14) was used with default settings to assort the 235,094 protein sequences into 7,690 gene phamilies, with the “--pangenome” flag so that PhaMMseqs would export a tab-separated values (TSV) file containing genome-to-pham-to-translation mappings used as the input file for PhamClust (Data Set S1).

### Pairwise genomic similarity calculations

PhamClust can use any of the following metrics to compute the similarity between a pair of genomes:

1. Jaccard coefficient (JC) (25) is the size of the set of intersecting phams in a pair of genomes divided by the size of the union of phams in those genomes. Neither gene length nor presence of paralogs is considered.
2. Gene content similarity (GCS) (4) is the bi-directional proportion of shared phams in a pair of genomes.

GCS = (2 × *P*_*S*_)/(*P*_1_ + *P*_2_),
where *P*_*S*_ is the number of phams shared by a pair of genomes, *P*_1_ is the number of phams in the query genome, and *P*_2_ is the number of phams in the target genome. Like JC, GCS does not account for gene length and paralogs.

1. POCP (15) is theoretically more accurate than GCS because it accounts for any paralogs present in either genome being compared. In paralog-free genomes, POCP is equivalent to GCS.

POCP = (*C*_1_ + *C*_2_)/(*T*_1_ + *T*_2_),
where *C*_1_ and *C*_2_ are the number of conserved proteins (including paralogs) in the query and target genomes, respectively. *T*_1_ and *T*_2_ are the total number of proteins in the query and target genomes, respectively. Gene length is not considered.

1. AF (16) is calculated as (*L*_*C*1_ + *L*_*C*2_)/(*L*_*T*1_ + *L*_*T*2_), where *L*_*C*1_ and *L*_*C*2_ are the summed lengths of conserved proteins in the query and target genomes, respectively. *L*_*T*1_ and *L*_*T*2_ are the summed lengths of all proteins in the query and target genomes, respectively.
2. AAI (19) is calculated as the length-weighted average percent identity between orthologous genes shared by a pair of genomes. Here, we modify the meaning to use Needleman-Wunsch global sequence alignments of orthologous gene pairs, rather than using two-way BLASTP alignments as in the original.
3. Proteomic equivalence (PEQ) is calculated as the product of AF and AAI. It thus accounts for both the proportion of shared genes between a pair of genomes, as well as the sequence identity within shared genes.

For each metric, the distance between a pair of genomes is calculated as 1.0 minus similarity(genome1, genome2). By default, PhamClust uses PEQ to derive pairwise genomic similarities/distances, as this metric reflects global genomic relatedness better than the others.

### Computing global sequence alignments

Needleman-Wunsch (26) global alignments were computed using the Python bindings for the PARASAIL alignment library (23). For any pair of sequences, the alignment was calculated using the BLOSUM62 substitution matrix (27) with affine gap penalties of −11/−1 for gap opening/extension, respectively. These are commonly used gap penalties for the BLOSUM62 matrix.

### Computing genome-wide nucleotide identity

Genome-wide BLASTN (28, 29) nucleotide identities were calculated for all pairs of mycobacteriophages in the data set. BLASTN was run between all pairs of genomes with an *E*-value cutoff of 1 × 10^−5^, culling limit of 1 (for example, to avoid double-counting degenerate repeat regions), and low-complexity masking disabled; resulting HSPs were processed to derive the total number of identical bases *N* found in all significantly similar regions for each pair of genomes. By summing the number of identical bases in HSPs from both source-to-target and target-to-source BLASTN searches and dividing by the summed genome lengths, a bi-directional genome-wide percent identity was obtained for each genome pair.

## RESULTS

### PhamClust workflow

**Fig 1:**
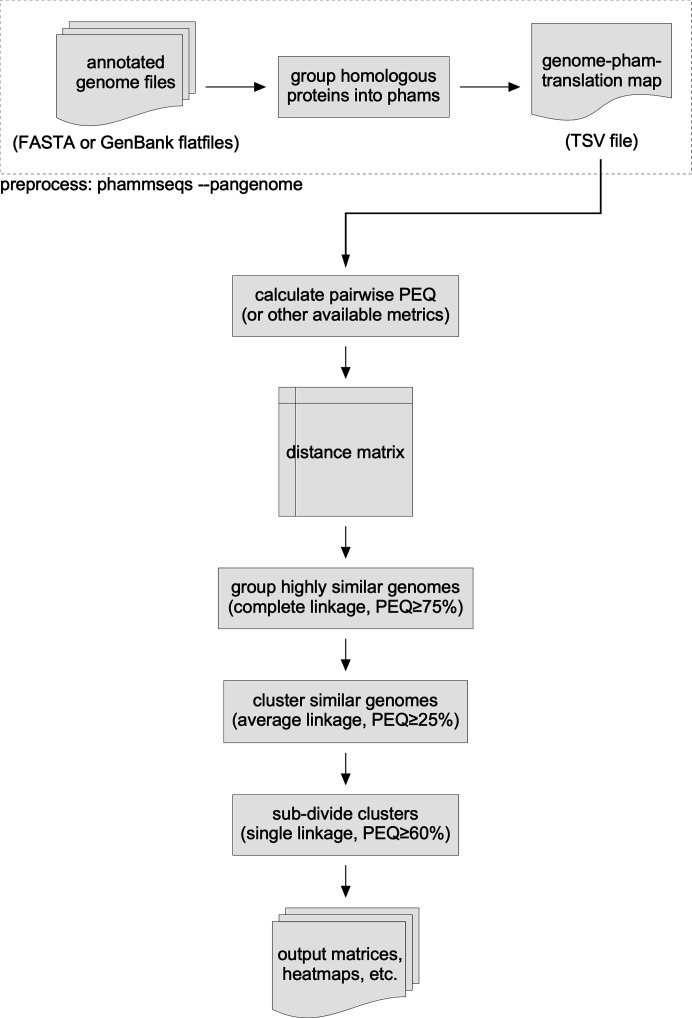
Workflow for PhamClust and genome comparisons. Annotated phage genomes can be compared by PhamClust following assembly of phage gene phamilies using PhaMMseqs, which can export a TSV file mapping genomes to phams to translations. PhamClust uses the data in this TSV file to calculate inter-genomic similarities (using PEQ by default) and populate a pairwise similarity matrix. The genomes are pre-clustered by complete linkage with similarity ≥75% to identify groups of highly homogeneous genomes; representatives of these groups are then clustered by average linkage with similarity ≥25% to form genome clusters. Non-singleton clusters can be optionally subclustered using single linkage with similarity ≥60%. For inter-genomic similarity calculations, five other metrics (JC, GCS, POCP, AF, and AAI) are available for use. For each of the clustering steps, users can specify different similarity thresholds and linkage types than those used by default.

The workflow for PhamClust begins with the identification of a set of annotated phage genomes for which the potential coding sequences have been identified. The predicted protein sequences are then sorted into groups of related “phamilies”’ using PhaMMseqs (30). PhaMMseqs records the genome-to-pham-to-translation mappings in a TSV file (e.g., Data Set S1), which is used as the input file for PhamClust. Genome names in the input file are assumed to be unique (which is true for the mycobacteriophage data set), and each genome’s pham-to-translation mappings are loaded into a set-like data structure that facilitates efficient comparison of genomes based on shared phams. Once genomes have been loaded into PhamClust, pairwise distance matrices can be calculated using various metrics (see Materials and Methods) although proteomic equivalence (PEQ) is the default function (Fig. 1). Pairwise distances can be calculated in parallel, allowing efficient comparison of thousands of phage genomes, with runtime scaling approximately linearly with the number of CPU cores utilized. The full distance matrix is hierarchically clustered to a user-specified distance threshold, and optionally subclustered if indicated. Cluster and subcluster matrices are written to a chosen output directory, along with heatmaps showing the relatedness of genomes within each non-singleton cluster (Fig. 1).

### Proteomic equivalence correlates strongly with genome-wide nucleotide identity

**Fig 2:**
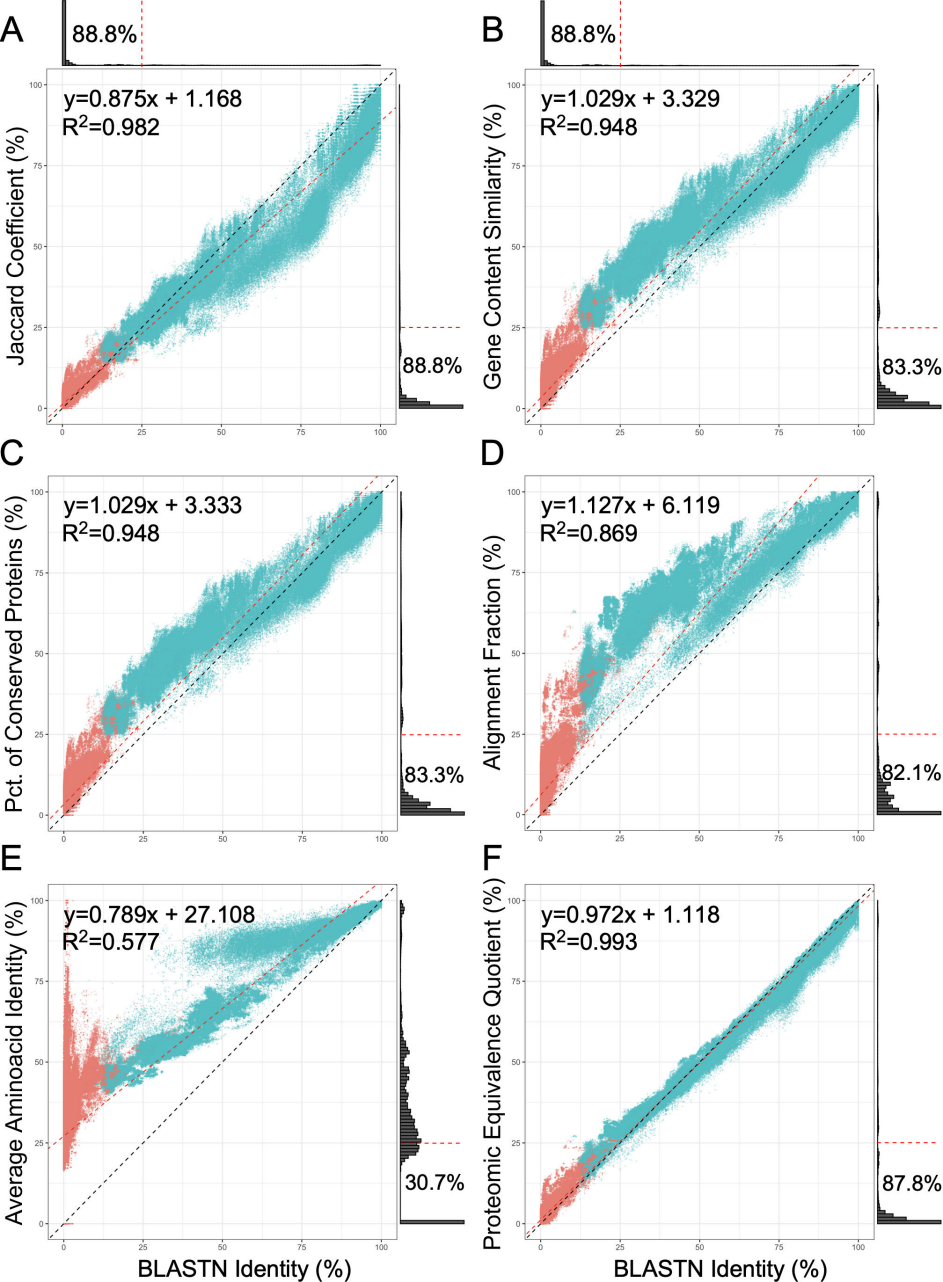
Scatterplots illustrating the relationships between protein-anchored genome similarity indices and genome-wide BLASTN identity. (A–F) Each dot represents a pair of mycobacteriophage genomes, colored teal if they have been manually assorted into the same genomic cluster, or red if they are in different clusters. The closer each metric correlates with BLASTN identity, the greater the number of comparisons that fall along the dashed black line on the diagonal of each subplot. For each subplot, the best fit linear model is shown (red dashed line) along with the line equation and Pearson *R^2^* value. The marginal histograms show that by far the most pairwise comparisons between phages show little or no detectable sequence similarity. The relationships between gNI and (A) JC, (B) GCS, (C) POCP, (D) AF, (E) AAI, (F) PEQ are shown.

PEQ is a metric designed to accommodate two key parameters of phage genome relationships: the number of shared genes and the extent of amino acid sequence similarity between the shared genes. It provides values between 1.0 and 0, where 1.0 reflects that all genes are shared and have 100% amino acid identity, and 0 indicates that there are no shared genes with amino acid sequence similarity greater than the detection threshold for pham assembly (equivalent to ~15% global amino acid identity with PhaMMseqs). A total of 2,248,260 data rows were obtained by pairwise comparison of all 2,121 mycobacteriophage genomes available at the time of the analysis (June 2022; Data Set S1). For every unique genome pair, the following measurements were made: JC, GCS, POCP, AF, AAI, proteomic equivalence (PEQ), and genome-wide nucleotide identity (gNI). We first compared the extant metrics of JC, GCS, POCP, AF, and AAI with gNI (Fig. 2A through E) and displayed the pairwise values as scatterplots. Most pairwise comparisons (88.8%) are between phages that have little or no nucleotide sequence similarity, and this tendency is reflected in the marginal histograms of each subplot, where the bulk of data points is found in the bottom left quadrant (Fig. 2).

Because phage genomes only rarely encode paralogous copies of genes, GCS and POCP values differ slightly for only some pairs of phages (Fig. 2B and C). These metrics have virtually indistinguishable relationships with gNI, and both consistently overestimate genome relatedness, especially as gNI decreases below 50% (Fig. 2B and C). Curiously, although mycobacteriophage clusters have ostensibly been grouped using a GCS threshold of 35% in the last several years, we observe large number of genome pairs in the same cluster that fall below this GCS threshold (as low as about 25%) (Fig. 2B). Many of these are likely historically entrenched and pre-date use of the 35% GCS threshold, while others may result from the fact that gene content-based distances do not satisfy triangle inequality. A bifurcating pattern is observed for AF (Fig. 2D) similar to the relationships previously described between gene content distances (1 minus GCS) and Mash distances (4, 31) and likely reflects differences in rates of horizontal gene transfer between different lineages of phages. AAI also shows a bifurcating pattern (Fig. 2E) which may result from different rates of mutation between genomes sharing either primarily vertically or primarily laterally inherited genes. Each of these five metrics has a non-linear relationship with gNI, complicating their usage for understanding genome relationships.

In contrast, proteomic equivalence (PEQ) has a roughly linear relationship with gNI (Fig. 2F). One notable departure is that when gNI is above 50%, PEQ tends to be slightly lower than gNI. This is likely a result of imperfect genome annotations with some open reading frames (ORFs) being omitted or being annotated with variable translational start codons. These will have a more adverse effect on PEQ when genomes are closely related than if they are more distantly related. When gNI is below 50%, PEQ tends to be slightly higher than gNI; in this case, PEQ likely provides a better estimate of global genome relatedness, because homologous but long-diverged proteins can be recognized even when nucleotide sequence similarities are no longer statistically significant. We note that computing the BLASTN values for this analysis required ~60 hours with 16 processing cores, whereas PEQ analyses took ~20 minutes using the same cores.

### PhamClust constructs phage clusters that mirror extant cluster assignments

To evaluate PhamClust outputs and to help calibrate PEQ thresholds, we compared PhamClust with a large set of mycobacteriophage genomes that have been assorted into clusters, subclusters, and singletons, using manual methods over a period of time. PEQ values can thus be established that largely mirror these extant groupings. However, we recognize that the extant cluster/subcluster assignments are likely imperfect in that they have been assembled stepwise by addition of phages to the data set over a 30-year period. Nonetheless, the extant structure is sound with mostly minor and known discrepancies. For example, Subcluster A1 phages share the general features of other Cluster A phages, but are more distantly related and could arguably constitute a new cluster. In general, changes as such have not been implemented to secure correspondence with the published literature. Nonetheless, investigation of the PEQ values that reconstitute the extant assignments is a useful starting point for evaluating PhamClust. From there, we can explore discrepancies between PhamClust and extant assignments and learn about the impact of PEQ values on the clustering.

**Fig 3:**
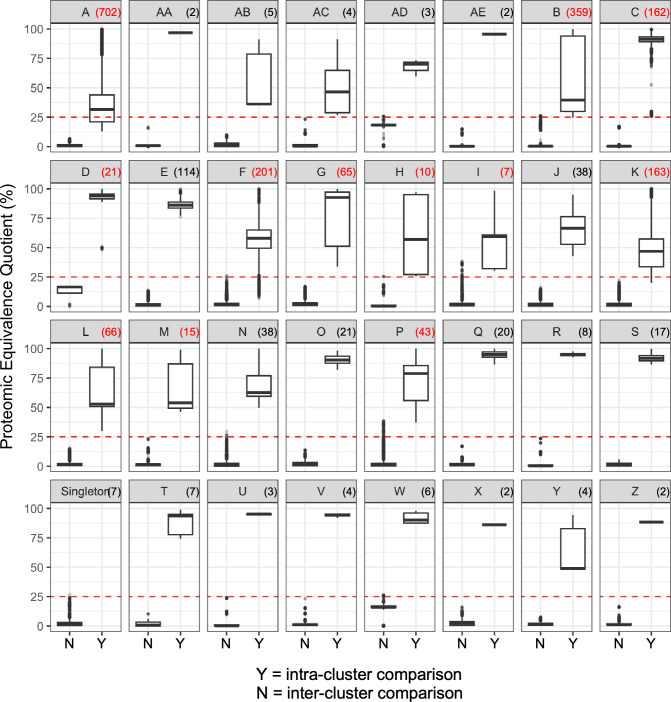
Mycobacteriophage clusters can be approximated by PEQ ≥ 25%. Each panel shows a box-and-whisker plot mapping inter-cluster (left) versus intra-cluster (right) PEQ distributions for genomes in a cluster. Box-and-whisker parameters use the R ggplot2 package default values: boxes correspond with the 25th through 75th quartiles with a line drawn at the median; whiskers above and below the boxes stretch to either a 1.5x of the interquartile range (IQR) or the min/max data point—whichever is closer to the box; outlying points above or below 1.5x IQR are also shown. The red dashed line is drawn at PEQ = 25%, the threshold which best differentiates between clusters. Cluster names are indicated in the title of each panel, with the number of genomes in each cluster in parentheses. Those clusters which have been further subclustered have the cluster size indicated in red text.

As a starting point, we found that a PEQ value of 25% differentiated well between intra-cluster and inter-cluster values (Fig. 3). However, initial efforts to cluster genomes down to this threshold resulted in the collapse of several clusters (P, I, and N; B and W; H and U) due to the existence of phages which clearly belong to one of these groups but also share extensive HGE-driven similarity to individual phages in another group. We thus used PhamClust to perform clustering in two iterations based on pairwise PEQ. In the first iteration, we hierarchically clustered genomes by complete linkage using a threshold of PEQ ≥ 75%. Hierarchical clustering proceeds in order from most-to-least related pairs of genomes, and complete linkage requires that as genomes are evaluated for entry into a cluster, they must meet or exceed the chosen similarity threshold with each of the extant members of the cluster in order to join the cluster. Clustering in this way results in homogeneous groups sharing most of their genes at high average percent identity; this homogeneity allows us to choose a good representative for merging related groups in the second iteration of clustering. The representative was chosen for each group by selecting the genome with the highest average pairwise PEQ to the remaining members of that group (i.e., the cluster medoid). Representatives were then hierarchically clustered with each other by average linkage using a threshold of PEQ ≥ 25%. By average linkage, a genome can only join a cluster if its mean similarity to all extant members (here again, genomes are added to clusters in descending order of similarity) of the cluster meets or exceeds the target threshold.

This two-step clustering workflow produced 33 clusters and 7 singletons, in close alignment to the extant groupings of 31 clusters and 7 singletons (Table 1). To compare the two, we assessed whether the PhamClust assignments agreed with extant cluster designations for each pairwise genome analysis. This yielded a confusion matrix (Table 1) from which PhamClust’s global precision (1.0) and sensitivity (0.734) could be calculated relative to the current assignments at PhagesDB.org (20). PhamClust’s overall output assignments are encouraging and indicate that PhamClust is not prone to clustering unrelated (or very distantly related) genomes. We note that the sensitivity appears to be low due to a high false negative rate (Table 1).

**TABLE 1:** Confusion matrix comparing PhagesDB clusters to PhamClust clusters

PhagesDB cluster	PhamClust cluster	Size	True positives^*a*^	True negatives^*b*^	False positives^*c*^	False negatives^*d*^
A1	4	189	149,094	498,069	0	96,957
A2–14,16–20	1	513	N/A^*e*^	N/A^*e*^	N/A^*e*^	N/A^*e*^
B	2	359	64,261	316,279	0	0
C	6	162	13,041	158,679	0	0
D	13	21	210	22,050	0	0
E	7	114	6,441	114,399	0	0
F1,3–5	3	194	18,742	192,960	0	1,358
F2	20	7	N/A^*e*^	N/A^*e*^	N/A^*e*^	N/A^*e*^
G	9	65	2,080	66,820	0	0
H	18	10	45	10,555	0	0
I	21	7	21	7,399	0	0
J	11	38	703	39,577	0	0
K	5	163	13,203	159,577	0	0
L	8	66	2,145	67,815	0	0
M	17	15	105	15,795	0	0
N	12	38	703	39,577	0	0
O	14	21	210	22,050	0	0
P	10	43	903	44,677	0	0
Q	15	20	190	21,010	0	0
R	19	8	28	8,452	0	0
S	16	17	136	17,884	0	0
T	22	7	21	7,399	0	0
U	28	3	3	3,177	0	0
V	25	4	6	4,234	0	0
W	23	6	15	6,345	0	0
X	30	2	1	2,119	0	0
Y	26	4	6	4,234	0	0
Z	31	2	1	2,119	0	0
AA	32	2	1	2,119	0	0
AB	24	5	10	5,290	0	0
AC	27	4	6	4,234	0	0
AD	29	3	3	3,177	0	0
AE	33	2	1	2,119	0	0
DS6A	DS6A	1	0	1,060	0	0
IdentityCrisis	IdentityCrisis	1	0	1,060	0	0
Kumao	Kumao	1	0	1,060	0	0
LilSpotty	LilSpotty	1	0	1,060	0	0
MalagasyRose	MalagasyRose	1	0	1,060	0	0
MooMoo	MooMoo	1	0	1,060	0	0
Sparky	Sparky	1	0	1,060	0	0
	Cumulative	2,121	272,335	1,877,610	0	98,315

^*a*^ Number of genome pairs correctly included in this group.

^*b*^ Number of genome pairs correctly left out of this group.

^*c*^ Number of genome pairs incorrectly included in this group.

^*d*^ Number of genome pairs incorrectly left out of this group.

^*e*^ Values are shared with the cell above.

### Disparities between PhamClust and manually derived genome clusters

Closer inspection revealed the sources of the two additional clusters assigned by PhamClust, relative to extant genome clusters. The first of these is an additional cluster containing all 189 of the genomes currently grouped in Subcluster A1. This change alone accounts for 96,957 (98.6%) of the false negative values from lost links between the other 513 phages in Cluster A (513 × 189 = 96,957). Subcluster A1 itself has good cohesion, with reasonably high average PEQ between members of this group (Fig. S2). However, members of Subcluster A1 share on average less than 25% PEQ with other Cluster A members and are therefore much more distantly related to them (Fig. S2). Reducing the PEQ threshold enough to merge Subcluster A1 with the rest of Cluster A results in a cascade of other cluster mergers whose end result bears little resemblance to the manual groupings. Overall, the inclusion of Subcluster A1 in Cluster A is inconsistent with other cluster assignments, although to retain association with previously reported analyses we do not propose formal re-assignment. As such, PhamClust has made suitable and appropriate assignments for the Subcluster A1 phages, and if not for the historical precedent, they would form a separate cluster. We note, however, that the extant Cluster A—including Subcluster A1—could be considered a supercluster as previously described (10). We also note that the relatedness of Subcluster A1 to the rest of Cluster A is similar to that between phages in Clusters B and W, between Clusters H and U, and among Clusters P, I, and N, which might also be considered superclusters in this scheme.

The remaining 1,386 false negatives (1.4%) are contributed by the second “extra” cluster generated by PhamClust, containing the seven genomes designated as Subcluster F2 (Table 1); this change affects links with the 194 remaining Cluster F phages (Fig. S3). Like the Subcluster A1 phages, the Subcluster F2 phages have good internal cohesion, but overall they are distantly related to other members of Cluster F. Both represent cases where PhamClust’s decision to leave the A1 and F2 phages out of Cluster A and Cluster F, respectively, is more consistent with level of intra-cluster cohesion observed in the remaining clusters at PhagesDB.

Comparison of all inter-cluster and intra-cluster PEQ values within each of the extant clusters illustrates the variety of relationships in different clusters (Fig. 3). For most clusters, the PEQ threshold value of 25% clearly separates the inter-cluster and intra-cluster values, consistent with PhamClust appropriately making these assignments. In a few instances, the inter-cluster distances include some comparisons that are about the PEQ = 25% threshold, notably in Clusters P, I, and N, which as noted above could conceivably meet the description of collectively forming a supercluster (Fig. 3). However, in only three extant clusters (Cluster A, Cluster F, and Cluster K) do the intra-clusters values fall below the PEQ = 25% threshold (Fig. 3), giving rise to the discrepancies discussed above. Most importantly, these comparisons illustrate that the relationships between phages within a cluster can be highly variable and cluster-specific. For example, there are similar number of Cluster C and Cluster K genomes, but the intra-cluster PEQ values are distinct both in average values and the variation (Fig. 3). Likewise, there are similar number of Cluster M and Cluster O phages, but their intra-cluster PEQ distributions are quite distinct (Fig. 3).

### Non-uniformity of cluster diversity precludes use of a “universal” subcluster threshold

**Fig 4:**
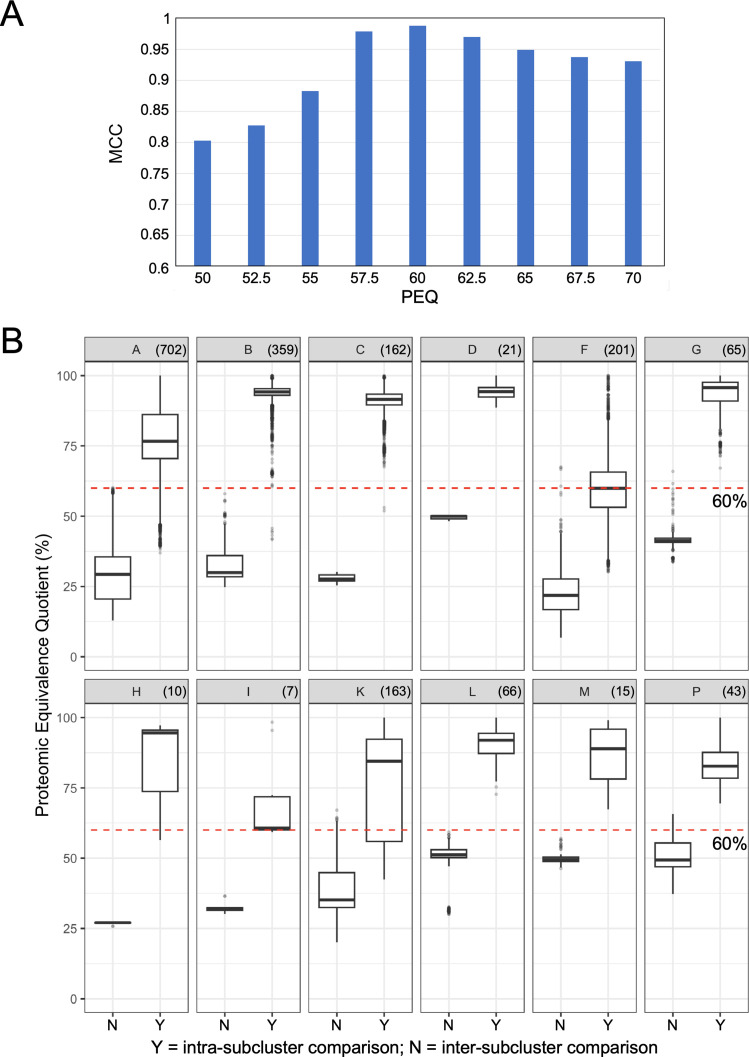
Mycobacteriophage subclusters can be approximated by PEQ ≥ 60%. (A) All of the extant mycobacteriophage clusters were subclustered by single-linkage with PEQ values in the range of 50% to 70% by steps of 2.5%. Resulting subclusters were compared to the extant subclusters and the MCC was calculated across all clusters per PEQ threshold to estimate how closely the resultant subcluster space globally compares with the extant space. MCC values range from −1 to 1, where 1 indicates that all edges perfectly agree with the reference data set, 0 indicates that the outcome was no better than random guessing, and −1 indicates that all edges perfectly disagree with the reference data set. (B) Each panel shows a box-and-whisker plot mapping inter-subcluster (left) versus intra-subcluster (right) PEQ distributions for genomes in a cluster. Cluster names are indicated in the title of each panel; box-and-whisker parameters are as described in Fig. 3. A red dashed line is drawn at PEQ = 60%, the threshold whose resultant subclusters most closely mirror the extant subcluster space (MCC = 0.98816).

To further explore these intra-cluster variations, we investigated how PEQ values can be used to compare PhamClust-derived subcluster designations relative to extant subcluster assignments. Twelve clusters (A, B, C, D, F, G, H, I, K, L, M, P) have extant divisions derived primarily from nucleotide similarities, and we examined inter-subcluster and intra-subcluster PEQ values for each of these clusters (Fig. 4). First, we explored the overall relationship between PEQ value and assortment of genomes into groups that align with extant subclusters. To do so, we used single-linkage clustering to subdivide each cluster using PEQ thresholds ranging from 50% to 70%; for each threshold, we calculated the Matthews correlation coefficient (MCC; unbiased accuracy score) between the predicted groups and extant subclusters (Fig. 4A; Table 2). Among the tested values, 60% PEQ gave the highest MCC score (0.9882), although this is likely weighted toward clusters with the largest number of genome members, and Clusters A, B, and C alone account for ~58% of phages in the data set.

**TABLE 2:** Outcomes from using the globally optimal PEQ threshold for subclustering

Cluster	Size	Expected^*a*^	Observed^*b*^	True positives*^c^*	True negatives^*f*^	False positives^*e*^	False negatives^*d*^	MCC^*g*^
A	702	19	19	38,907	206,942	101	101	0.9969
B	359	13	14	29,807	34,448	0	6	0.9998
C	162	2	3	12,562	477	0	2	0.9978
D	21	2	2	190	20	0	0	1
E	114	1	1	6,441	0	0	0	N/A
F	201	6	8	17,593	2,310	6	191	0.9545
G	65	5	4	1,547	531	2	0	0.9975
H	10	2	3	22	16	0	7	0.7265
I	7	2	3	10	10	0	1	0.9091
J	38	1	1	703	0	0	0	N/A
K	163	7	10	3,529	8,262	77	1,335	0.7755
L	66	5	5	638	1,507	0	0	1
M	15	3	3	43	62	0	0	1
N	38	1	1	703	0	0	0	N/A
O	21	1	1	210	0	0	0	N/A
P	43	6	4	632	231	40	0	0.8954
Q	20	1	1	190	0	0	0	N/A
R	8	1	1	28	0	0	0	N/A
S	17	1	1	136	0	0	0	N/A
T	7	1	1	21	0	0	0	N/A
U	3	1	1	3	0	0	0	N/A
V	4	1	1	6	0	0	0	N/A
W	6	1	1	15	0	0	0	N/A
X	2	1	1	1	0	0	0	N/A
Y	4	1	2	2	0	0	4	N/A
Z	2	1	1	1	0	0	0	N/A
AA	2	1	1	1	0	0	0	N/A
AB	5	1	3	3	0	0	7	N/A
AC	4	1	2	3	0	0	3	N/A
AD	3	1	1	3	0	0	0	N/A
AE	2	1	1	1	0	0	0	N/A
			Cumulative	113,951	254,816	226	1,657	0.9882

^*a*^ Extant number of subclusters as found in PhagesDB.

^*b*^ Observed number of subclustering when using PhamClust with PEQ ≥ 60% by single-linkage.

^*c*^ Number of genome pairs in the same subcluster in PhamClust output and at PhagesDB.

^*d*^ Number of genome pairs in different subclusters in PhamClust and at PhagesDB.

^*e*^ Number of genome pairs in the same subcluster in PhamClust output but not at PhagesDB.

^*f*^ Number of genome pairs in different subclusters in PhamClust output but not at PhagesDB.

^*g*^ Matthews correlation coefficient (unbiased accuracy score): −1 indicates that every pair of genomes was mis-classified, while 1 means every pair of genomes was correctly classified.

A closer inspection of the impact of PEQ on subdivisions within clusters shows that although the 60% PEQ value discriminates well between inter-subcluster and intra-subcluster groups for many clusters, some clusters split at different PEQ values (Fig. 4B; Table 2). For example, a 60% PEQ threshold does relatively poorly at alignment of subdivisions in Clusters H and K, and only somewhat better for Clusters I and P (Table 2). For Clusters H, I, and K, the PEQ values giving optimal alignment are below the 60% PEQ threshold, whereas for Cluster P it is above the 60% PEQ cutoff value (Table S1).

These observations suggest that there is no single PEQ threshold value that replicates our previous subdivision of clusters. This is further illustrated by comparing the mean inter-subcluster and intra-subcluster PEQ values and their distributions (Fig. 4B). For clusters such as A, F, and K, the distributions overlap, and as noted above, PEQ values of 50% and 57% are better discriminators for Clusters F and K (Fig. 4B). For Cluster A, the 19 extant subclusters are generally well defined by PhamClust, but Subcluster A2 is notably diverse (Fig. S2) and includes some low intra-subcluster PEQ values (Fig. 4B; Fig. S2). Subcluster A2 could arguably be divided into separate subclusters. In contrast, Cluster F appears to have very little natural partitioning (Fig. S3) and the members of Subcluster F1 show a similar range of pairwise PEQ values as is seen across the entirety of Cluster A, although these appear as a continuum of values rather than discrete partitions. Cluster K likewise has relatively poor discrimination of inter-subcluster and intra-subcluster PEQ values (Fig. 4B; Fig. S4), and the subcluster divisions are less clear; some subclusters (e.g., K1 and K6) could arguably be further divided. One Subcluster K4 phage (Boilgate) has been reassigned to a new subcluster (K8) as a result of PhamClust’s output.

**Fig 5:**
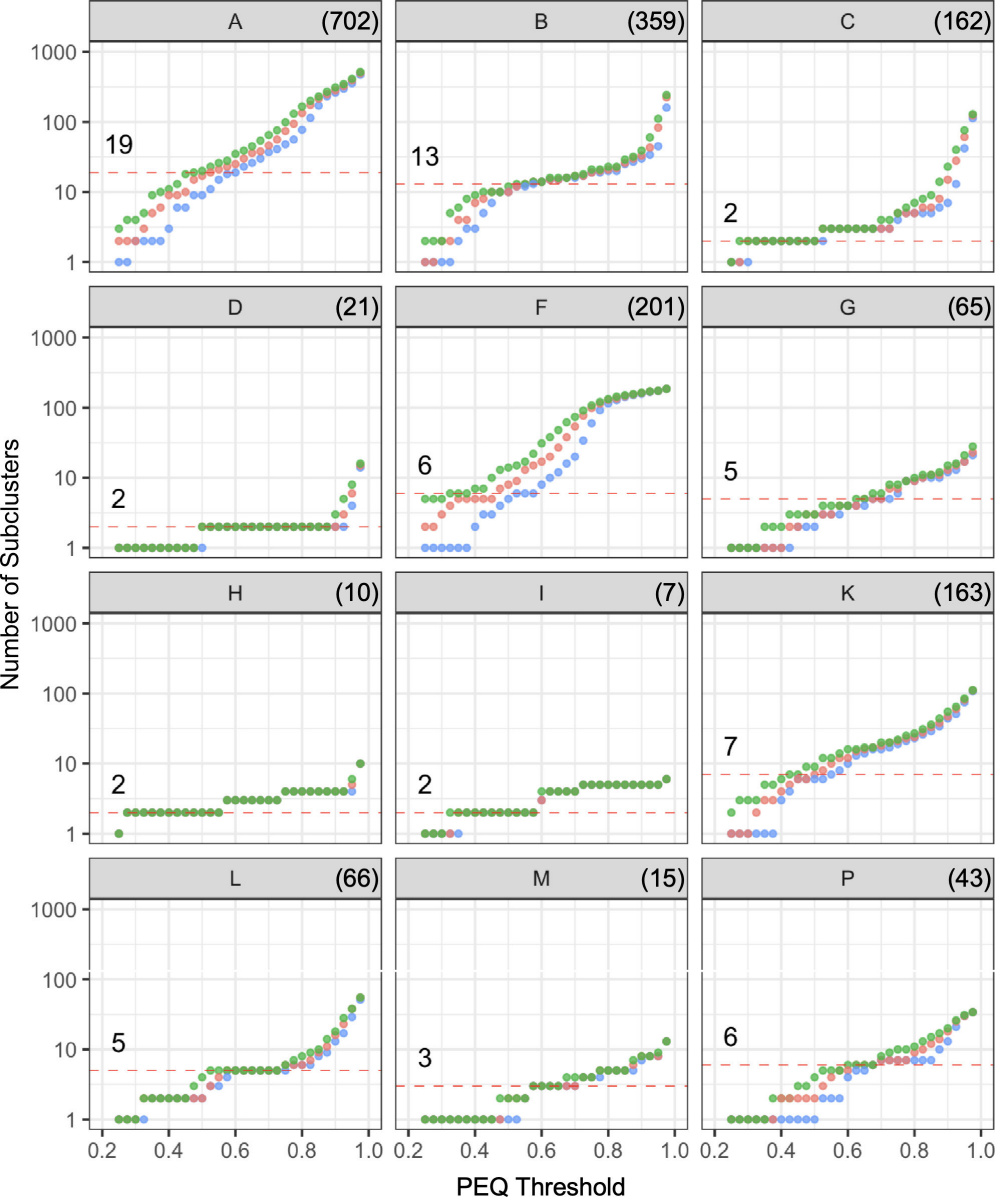
Mycobacteriophage clusters show non-uniform heterogeneity. Each panel shows the number of subclusters resulting from each PEQ threshold by single-linkage (blue), average-linkage (red), or complete-linkage (green) in each of the clusters which have been manually subclustered. A red dashed line indicates the current number of manually determined subclusters for each cluster. For each cluster, there exists at least one PEQ/linkage pair producing the same number of subclusters as has been determined manually. For most clusters, the membership of these subclusters is not markedly different from the manually determined subclusters.

It is plausible that the variation in cluster subdivisions and the correlations with extant subcluster groups could arise from misplacement of genomes in the manual assemblies, rather than from inherent differences in intra-cluster relationships. We therefore examined PhamClust outputs that are agnostic to the extant groupings (Fig. 5). To do so, we used single-linkage, average-linkage, and complete-linkage PEQ values to determine the relationships between PEQ values and the number of subclusters formed; this was calculated for the 12 divided clusters from PEQ values of 25% to 97.5% in steps of 2.5% (Fig. 5). Although the extant number of subclusters generally mirror grouping using single-linkage PEQ values between 0.5 and 0.7 (Fig. 5), the relationships differ greatly among clusters. For example, for some clusters, there is a substantial separation of the patterns for the three different linkage methods. It is striking, for example, that at the 60% PEQ value, the three linkage methods give the same number of subdivisions for Cluster B, but for Cluster F at the 60% PEQ value the number of subdivisions differ by more than 10-fold for different linkage methods (Fig. 5). Furthermore, some clusters (e.g., Cluster B) have a seemingly sigmoidal relationship between PEQ and the number of subclusters formed, whereas for others (e.g., Cluster A) this is much less evident. There is thus no universal set of relationships within groups of related phage genomes (i.e., clusters).

### Clustering the phage RefSeq data set using PhamClust

Because PhamClust can quickly and rapidly assemble the complex mycobacteriophage genomes, it seems likely that it could be readily applied to other phage genome collections. To explore this, we used PhamClust to assort genomes in the GenBank RefSeq phage collection, using PEQ threshold values of 25% and 60% for cluster and subcluster groupings, respectively (Data Set S2). The 4,981 genomes were assembled into a total of 884 groups, of which 50% (441) are multi-genome clusters and 50% are unclustered genomes (i.e., singletons). To assess the validity of the output, we manually compared the groupings with those reported previously for a set of phage genomes of the *Enterobacteriaceae* (10). At the cluster level, we were able to map 53 of the 56 previously defined clusters to genomes within the RefSeq data set. Of these, 52 (98%) mapped precisely to PhamClust-predicted groups, illustrating excellent agreement. Only one of the extant clusters shows disagreement, the T4-like phages, described as the Lytic2 group in reference (10). Two phages (Lw1 and RB43) both assigned to Subcluster Lytic2I (10) map in a different PhamClust group (#171) than the other phages (#8) (Data Set S2). However, this is unsurprising as these have very low levels of similarity to other Lytic2 phages as shown by nucleotide dotplot and other analyses (10). There is reasonably good agreement between the PhamClust output and the subcluster groupings of Grose and Casjens (10), but the alignments differ among clusters, reflecting the cluster variations reported previously (10), and mirroring our observations with the mycobacteriophages shown above.

## DISCUSSION

We have described here a new bioinformatic tool for investigating phage genome relationships and constructing groupings of related phages. PhamClust compares genomes based on their PEQ, a genome similarity metric that accounts for both the proportion of genes shared between genomes and the evolutionary distances of the shared genes based on amino acid sequence similarity. PEQ values are determined for each pair of genomes in a data set, and the genomes can then be assorted into groups using either single-linkage, average-linkage, or complete-linkage methods. When PhamClust is used to sort 2,121 mycobacteriophages into groups using an average-linkage threshold of 25% PEQ, it closely recapitulates the extant set of “clusters”. The minor differences arise from historically fixed idiosyncrasies in extant cluster assignments and not from errant assignments made by PhamClust.

Comparing phage genomes using PhamClust requires a three-step process. The first is to use the previously described algorithm “PhaMMseqs” to assemble the protein sequences of annotated ORFs into phamilies of related genes, yielding a data set that maps the genomes, phamilies, and amino acid sequences. This is then used as input data for PhamClust that assigns PEQ values for every pairwise genome comparison. Finally, PhamClust uses these PEQ values to form clusters of related genomes with PEQ cutoff as a user-defined variable (Fig. 1).

Comparison of the PEQ values produced by PhamClust with other methods for genome comparisons suggests that it more closely mirrors overall nucleotide sequence similarity than using Jaccard coefficient, gene content similarity, percentage of conserved proteins, the aligned fraction, or average amino acid identity. This is notable as VIRIDIC (11), which assigns phages to genus- and species-level groups, uses a nucleotide sequence similarity approach that is computationally demanding. In contrast, PhamClust (coupled with PhaMMseqs) is computationally efficient and can process relatively large sets of phage genomes. In our hands, VIRIDIC struggled to analyze more than half of the ~2,100 mycobacteriophage genomes examined in this study, suggesting it is likely not scalable to all current phage entries in the ReqSeq database (~5,000). In contrast, we have previously demonstrated that PhaMMseqs can be used to quickly and accurately perform pham assembly for all RefSeq genomes, and here we showed that the combination of PhaMMseqs and PhamClust is readily scaled to ~2,100 phage genomes; we have similarly used it for comparative analysis of ~4,300 phage and prophage genomes (32). PhamClust was also able to analyze the collection of nearly 5,000 RefSeq phage genomes, requiring only 2.5 hours of runtime on a laptop with eight cores (16 threads) and with the resulting clusters closely mirroring published expert-curated groupings, for the two largest groups of phages (*Enterobacteriaceae* and *Mycobacterium* phages) for which extant groupings are available. PhamClust may thus be a broadly useful tool for phage taxonomy.

An underlying challenge to forming groupings of phages based on sequence relatedness is that the genomes are pervasively mosaic resulting from horizontal genetic exchange (2). These HGE events do not require extensive sequence homology and there are few constraints as to which genomes participate in genetic exchange (2). Any grouping of genomes according to their relatedness thus likely reflects differences in environmental prevalence and isolation biases rather than hard biological divisions resulting from barriers to exchange (6). However, this is revealed most clearly by comparison of a large number of genomes of phages isolated on a single common host strain of bacteria, and the mycobacteriophages form a unique data set in this regard. Nonetheless, related groups of phages can be constructed using PhamClust, and the description of these groups (e.g., “Clusters”, “Families”, etc.) is a user decision. However, we predict that when much larger phage sequence data sets are available, the division into major groupings will become more challenging, and that a universal PEQ value is less justifiable. This is thus expected to mirror the current situation encountered when examining individual clusters to see if subdivision is warranted. PhamClust clearly shows that some mycobacteriophage clusters warrant subdivision based on current parameters, but the relationships between genomes are qualitatively different in different clusters. As such, an intra-cluster PEQ threshold of value of 60% can be used as a starting point for exploring cluster subdivisions, but then each cluster warrants further analysis and subsequent adjustments.

PhamClust provides a useful analytic tool for exploring this heterogeneous aspect of phage genome relationships. Interestingly, the number of subclusters formed varies with PEQ value, but the relationships are cluster-specific. For example, because of the sigmoidal-like relationship in Cluster B phages (Fig. 5), the number of subclusters varies little over a span of PEQ values from 50% to 70%. In contrast, for Cluster A phages, the number of potential subclusters varies about fivefold over a similar PEQ span. The reasons for these differences are interesting but not yet clear, and it is likely that phage lifestyle, host environments, phage host range, phage-encoded recombination systems, variability in host range mutability, and phage-encoded DNA replication systems, all play important roles.

## Data Availability

PhamClust is available from GitHub (https://github.com/chg60/phamclust) or installed from PyPI (https://pypi.org/project/phamclust/). The PhaMMseqs package is available at PyPI (https://pypi.org/project/phammseqs/) and GitHub (https://github.com/chg60/PhaMMseqs.git). Databases are available at http://databases.hatfull.org. All phage genomes are available at GenBank and at https://phagesdb.org.

## SUPPLEMENTAL MATERIAL

Data Set S1 msystems.00443-23-s0001.xlsx A tab-separated values (TSV) file produced by PhaMMseqs for the 2,121 genomes analyzed in this study.

Data Set S2 msystems.00443-23-s0002.xlsx A set of 4,981 complete phage genome annotations retrieved from RefSeq and analyzed with PhaMMseqs and PhamClust.

Supplemental information msystems.00443-23-s0003.pdf Supplemental table and figures, as well as captions for Data Sets S1 and S2.

ASM does not own the copyrights to Supplemental Material that may be linked to, or accessed through, an article. The authors have granted ASM a non-exclusive, world-wide license to publish the Supplemental Material files. Please contact the corresponding author directly for reuse.
