# Supplementary material for: PhamClust: a phage genome clustering tool using proteomic equivalence
Source: mSystems. 2023 Oct 4;8(5):e00443-23. doi: 10.1128/msystems.00443-23 (PMC10654103; doi:10.1128/msystems.00443-23)
Supplement: Supplemental information — Supplemental table and figures, as well as captions for Data Sets S1 and S2. [file msystems.00443-23-s0003.pdf]

Supplementary Information

Table S1. Cluster-specific PEQ ranges giving the minimum number of differences from extant subcluster groupings.

Data Set S1. A tab-separated values (TSV) file produced by PhaMMseqs for the 2121 genomes analyzed in this study. This is used as the primary input file by PhamClust.

Data Set S2. A set of 4981 complete phage genome annotations were retrieved from RefSeq and analyzed with PhaMMseqs (for pham assembly) and PhamClust (for genome clustering), both with default runtime parameters. Accession number and name are shown for each phage together with the Cluster and Subcluster assigned using randomly assigned numbers as Cluster/Subcluster names. i.e. *Escherichia* phage ADB-2 is sorted into Cluster #6, and in Subcluster #3 within Cluster #6. The PEQtoCluRepr value is also shown which is the PEQ value for each phage shown relative to the 'representative' cluster member chosen by PhamClust (see Methods). A high value for PEQtoCluRepr reflect close similarity to the representative member; a low values for PEQtoCluRepr reflects a distant relative to the representative member. For the RefSeq *Enterobacteriaceae* phages included in the analysis of Grose and Casjens (10) their cluster (e.g. Lytic1, Lytic2; Temperate1, Tempertate2 etc) and subcluster (e.g. Lytic 1A, Lytic1B etc) assignments are also shown. Color sharing reflects phage groups with the same extant cluster assignment.

Table S1. Cluster-specific PEQ ranges giving the minimum number of deviations from extant subcluster groupings.

Cluster	Size[1]	PEQ Range[2]	Expect[3]	Observe[4]	TP[5]	FN[6]	FP[7]	TN[8]	MCC[9]
A	702	60	19	19	38907	101	101	206942	0.9969
B	359	60	13	14	29807	6	0	34448	0.9998
C	162	32.5-52.5	2	2	12564	0	0	477	1
D	21	52.5.92.5	2	2	190	0	0	20	1
E	114	25-87.5	1	1	6441	0	0	0	N/A
F	201	50	6	5	17784	0	6	2310	0.9985
G	65	67.5-72.5	5	5	1547	0	0	533	1
H	10	27.5-55	2	2	29	0	0	16	1
I	7	37.5-57.5	2	2	11	0	0	10	1
J	38	25-62.5	1	1	703	0	0	0	N/A
K	163	57.5	7	8	4238	626	126	8213	0.8780
L	66	60-75	5	5	638	0	0	1507	1
M	15	57.5-70	3	3	43	0	0	62	1
N	38	25-65	1	1	703	0	0	0	N/A
O	21	25-90	1	1	210	0	0	0	N/A
P	43	67.5	6	6	632	0	0	271	1
Q	20	25-90	1	1	190	0	0	0	N/A
R	8	25-95	1	1	28	0	0	0	N/A
S	17	25-92.5	1	1	136	0	0	0	N/A
T	7	25-77.5	1	1	21	0	0	0	N/A
U	3	25-95	1	1	3	0	0	0	N/A
V	4	25-92.5	1	1	6	0	0	0	N/A
W	6	25-90	1	1	15	0	0	0	N/A
X	2	25-85	1	1	1	0	0	0	N/A
Y	4	25-47.5	1	1	6	0	0	0	N/A
Z	2	25-87.5	1	1	1	0	0	0	N/A
AA	2	25-95	1	1	1	0	0	0	N/A
AB	5	25-35	1	1	10	0	0	0	N/A
AC	4	25-27.5	1	1	6	0	0	0	N/A
AD	3	25-67.5	1	1	3	0	0	0	N/A
AE	2	25-95	1	1	1	0	0	0	N/A
**Cumul**.	2114	N/A	91	92	114875	733	233	254809	0.9939

[1]Number of genomes in the cluster
[2]Range of PEQ values giving the minimum number of deviations (FN + FP) from extant subclusters
[3]Extant number of subclusters in this cluster
[4]Number of subclusters obtained using a PEQ threshold in the indicated range
[5]Number of genome pairs correctly subclustered together
[6]Number of genome pairs incorrectly not subclustered together
[7]Number of genome pairs incorrectly subclustered together
[8]Number of genome pairs correctly not subclustered together
[9]Matthews Correlation Coefficient (unbiased accuracy score); -1 indicates that every pair of genomes was mis-classified, while 1 means every pair of genomes was correctly classified

Supplementary Figure Legends

Figure S1. Mycobacteriophage undivided clusters show non-uniform heterogeneity. Each panel shows the number of subclusters resulting from a range of PEQ thresholds by single-linkage (blue), average-linkage (red), or complete-linkage (green) in each of the clusters which have been manually subclustered. A red dashed line indicates the current number of manually determined subclusters for each cluster (1, as none of these clusters has been sub-divided). For each cluster, there exists at least one PEQ/linkage pair producing the same number of subclusters as has been determined manually. For most clusters, the membership of these subclusters is not markedly different from the manually determined subclusters.

Figure S2. Heatmap of Cluster A pairwise PEQ values. Pairwise PEQ values were calculated between all 702 genomes in mycobacteriophage Cluster A. These values were plotted as a heatmap following a 3-point color gradient: red=0%, yellow=50%, and green=100%. Extant subcluster boundaries are indicated along the y-axis.

Figure S3. Heatmap of Cluster F pairwise PEQ values. Pairwise PEQ values were calculated between all 201 genomes in mycobacteriophage Cluster F. These values were plotted as a heatmap following a 3-point color gradient: red=0%, yellow=50%, and green=100%. Extant subcluster boundaries are indicated along the y-axis.

Figure S4. Heatmap of Cluster K pairwise PEQ values. Pairwise PEQ values were calculated between all 201 genomes in mycobacteriophage Cluster K. These values were plotted as a heatmap following a 3-point color gradient: red=0%, yellow=50%, and green=100%. Extant subcluster boundaries are indicated along the y-axis.

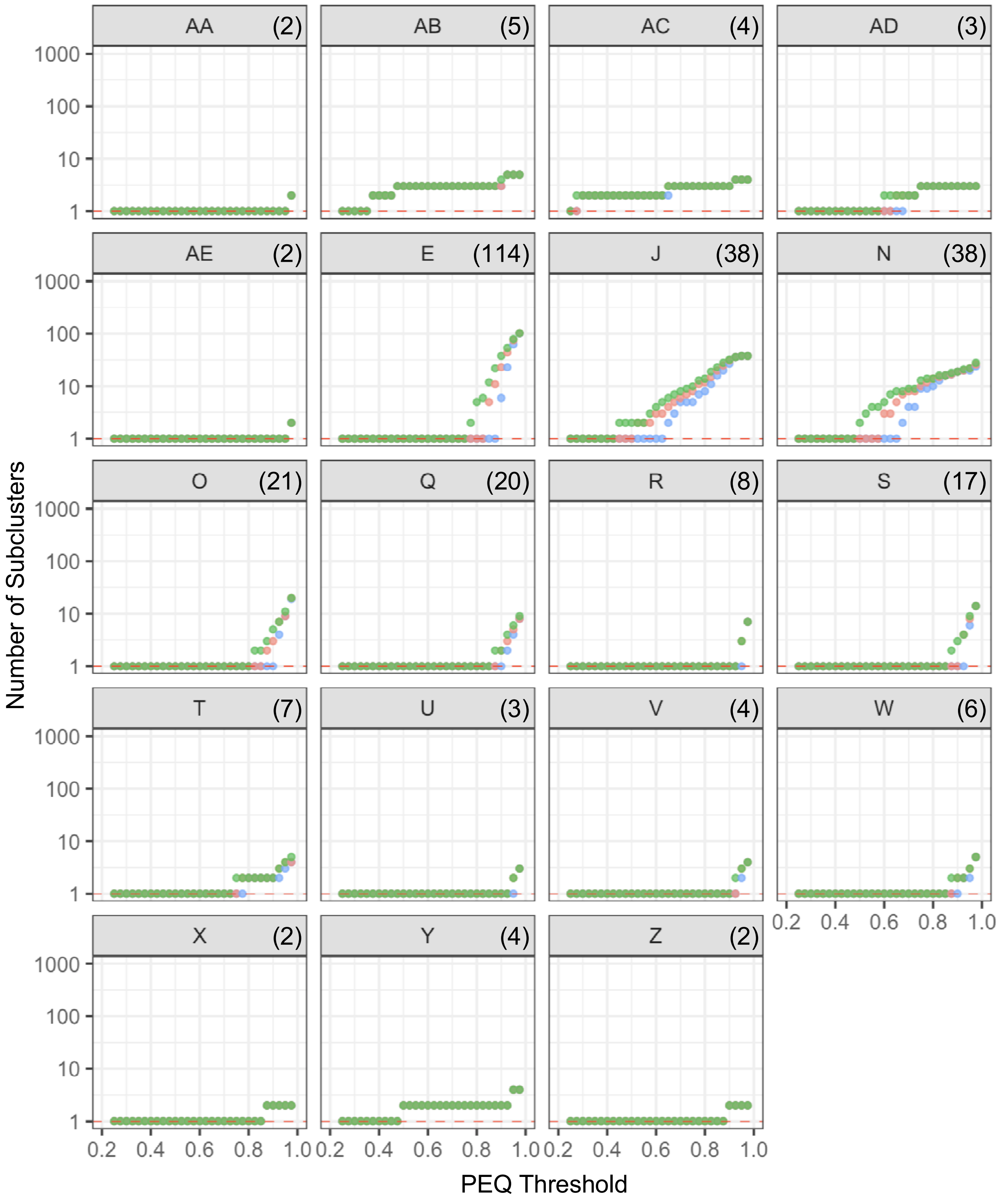
AA (2)
AB (5)
AC (4)
AD (3)
AE (2)
E (114)
J (38)
N (38)
O (21)
Q (20)
R (8)
S (17)
T (7)
U (3)
V (4)
W (6)
X (2)
Y (4)
Z (2)
1000
100
10
1
0.2 0.4 0.6 0.8 1.0
Number of Subclusters
PEQ Threshold

Figure S1

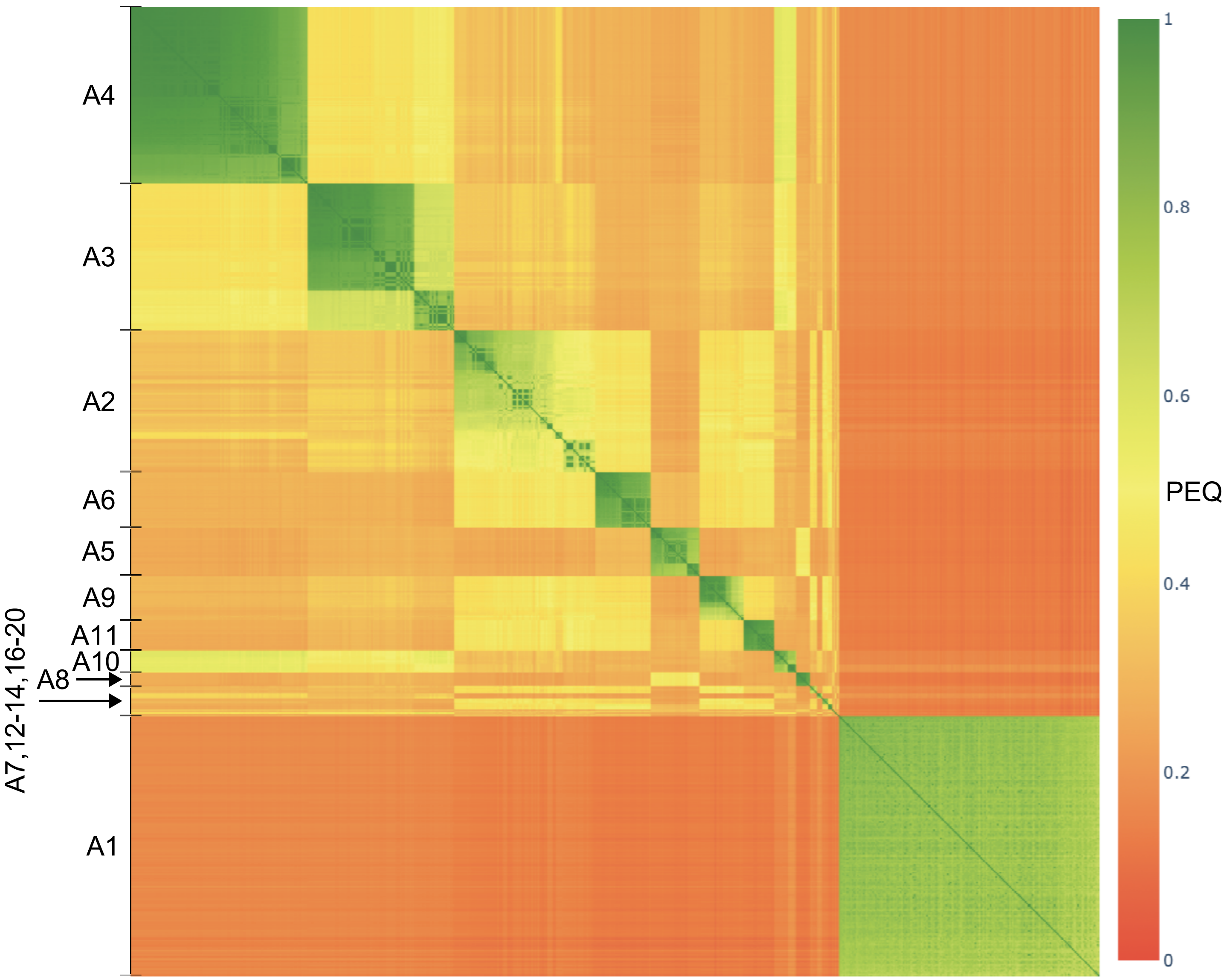

Figure S2

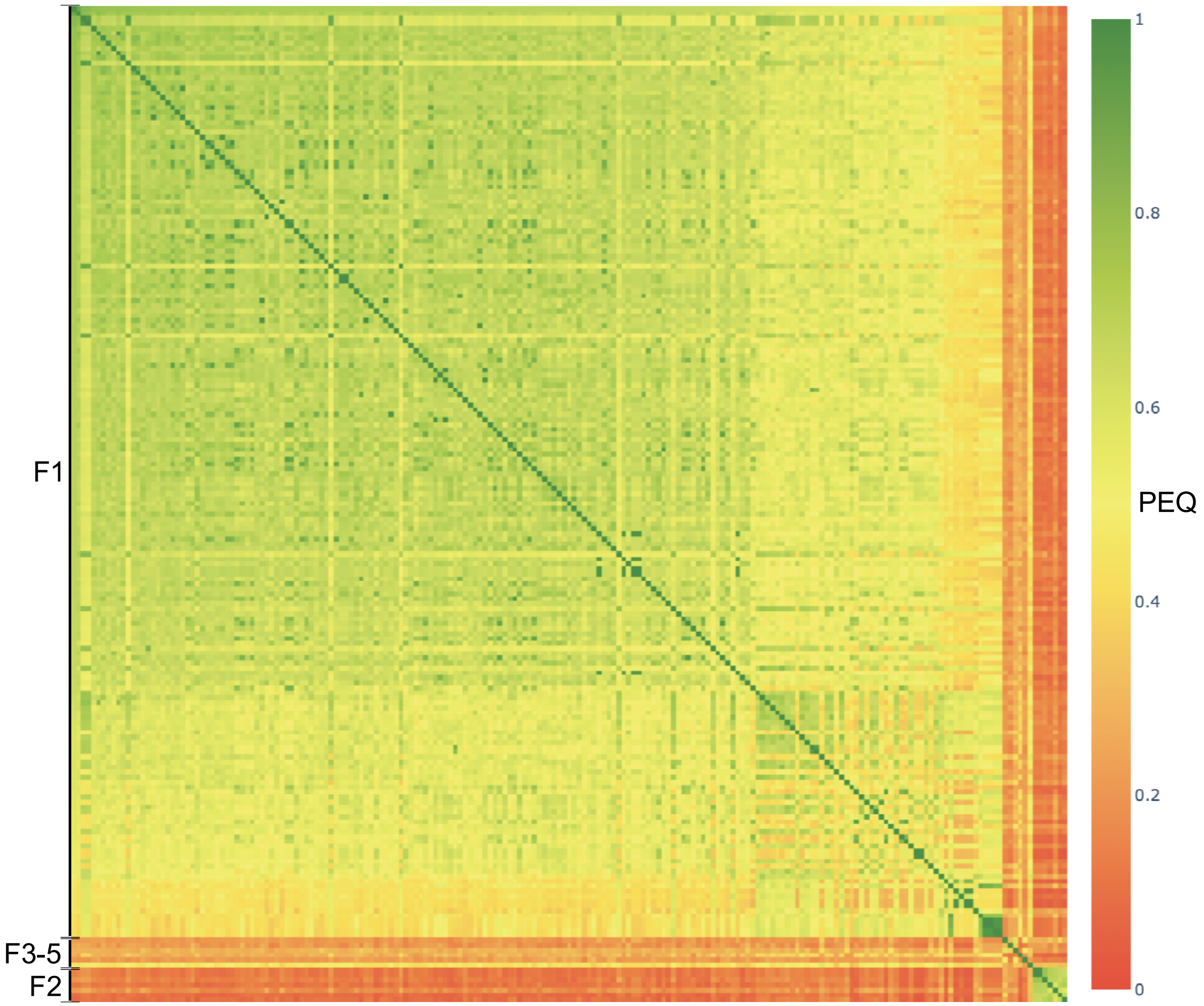

Figure S3

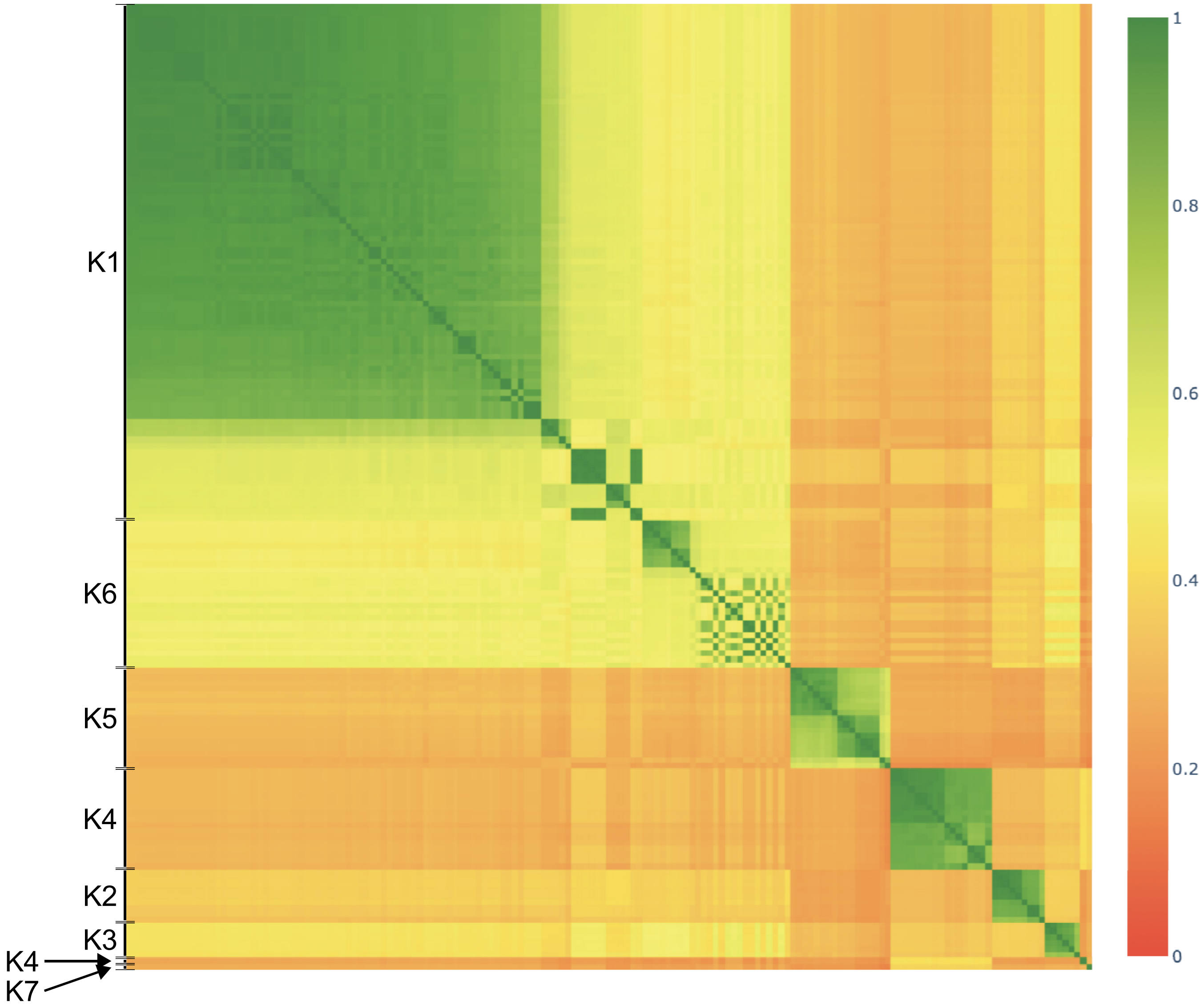

Figure S4
